# Supplementary material for: The dependence of children’s generalization on episodic memory varies with age and level of abstraction
Source: Nat Commun. 2025 Oct 7;16:8894. doi: 10.1038/s41467-025-63934-w (PMC12504698; doi:10.1038/s41467-025-63934-w)
Supplement: Supplementary file 1 — Supplementary Information [file 41467_2025_63934_MOESM1_ESM.pdf]

Supplementary Information

Supplementary Note 1

Age and Typicality on Species Class Category Knowledge

We tested whether species-class category knowledge improved with age or differed between typical versus atypical species, we conducted the following linear model:

lmer(species-class category knowledge accuracy ~ age * typicality + (1 | participant)

Species class knowledge was positively associated with age (β= 0.006, *SE*= 0.009, *t*(287.1)= 7.29, *p*< .001) (see Figure S2). There was no statistically significant interaction with typicality (β= -0.02, *SE*= 0.011, *t*(845)= -1.86, *p*= .06). It is also important to note that the younger children in our sample, aged 3-5 years (*M*= 52.79 months, range= 36-71), performed significantly above chance on this task, *M*= .72, *SD*= .22, *t*(60)= 7.84, *p*<.001.

Supplementary Note 2

Correlations between Memory Tasks

We asked whether performances on the 4 generalization tasks and memory precision on the animal-location recall task correlated with each other when controlling for age. We found that after partialling out the effects of age, generalization performances the New Animals, New Places, and Baby Animals tasks positively correlated with each other (New Animals vs. New Places: β= 0.81, *SE*= 0.06, *t*(188) = 13.19, *p* <.001, 95% CI [0.69, 0.93], New Animals vs. Baby Animals, β= 0.96, *SE*= 0.05, *t*(188) = 20.85, *p* <.001, 95% CI [0.87, 1.05], and New Places vs. Baby Animals, β= 0.81, *SE*= 0.06, *t*(188) = 13.87, *p* <.001, 95% CI [0.69, 0.92], Each of these tasks also correlates with displacement errors on the animal-location recall task (all *p*< .001). Performance on the New Species task showed no statistically significant correlation to any of the other 3 generalization tasks, all *p*s> .64, or with memory precision, *p*=

.96. In sum, we did not find evidence for statistically significant associations between generalization performance on the New Species task and memory specificity or even to any other generalization tasks on the inter-individual differences level.

Supplementary Note 3

Contingencies of Generalization on Regional Accuracy

We conducted a similar set of analyses as described in Results section 2.1. to test whether generalization is contingent on memory specificity but using regional accuracy score instead of displacement error. The results were the same as those reported in the Results section 2.1., in which the displacement error was a fixed effect.

For the New Animals generalization task, generalization accuracy was associated with verbal intelligence, $\beta = 0.30$, $SE = 0.15$, $z = 2.07$, $p = .039$, 95% CI [0.02, 0.60], age, $\beta = 1.07$, $SE = 0.20$, $z = 5.30$, $p < .001$, 95% CI [0.69, 1.49], regional accuracy, $\beta = 1.51$, $SE = 0.21$, $z = 7.30$, $p < .001$, 95% CI [1.12, 1.94], and an age*displacement error interaction, $\beta = 0.59$, $SE = 0.16$, $z = 3.83$, $p < .001$, 95% CI [0.29, 0.91]. The likelihood of correctly generalizing that a new animal exemplar would go to the place as its same-species members depended on children's memory accuracy of animal-location associations for all children. However, the degree of this contingency varied with age: older children showed a stronger tie between generalization and memory accuracy compared to younger children. Nonetheless, younger children's (ages 3-5) generalization accuracy was significantly associated with regional accuracy, $\beta = -1.01$, SE= 0.15, z= -6.74, $p < .001$, 95% CI [-1.31, -0.72].

We observed similar results for the New Places generalization task. Generalization accuracy was associated with age, $\beta = 1.00$, SE= 0.19, z= 5.25, $p < .001$, 95% CI [0.64, 1.40], regional accuracy, $\beta = 1.82$, SE= 0.19, z= 9.49, $p < .001$, 95% CI [1.46, 2.22], and an

age*regional accuracy interaction, β= 0.84, SE= 0.15, z= 5.54, p< .001, 95% CI [0.55, 1.16]. There was no statistically significant effect of verbal intelligence, β= 0.25, SE= 0.14, z= 1.82, p= .069, 95% CI [-0.02, 0.53]. The likelihood of correctly generalizing that a given learned animal would go to a new but similar place was contingent on children's memory accuracy of the animal-location associations. Again, the degree of this contingency increased as children's age increased. Nonetheless, younger children's generalization accuracy was contingent on their regional accuracy β= 1.33, SE= 0.16, z= 8.46, p< .001, 95% CI [1.04, 1.66].

For the Baby Animals generalization task, we found the same pattern: generalization accuracy was associated with age, β= 1.56, SE= 0.22, z= 7.16, p< .001, 95% CI [1.16, 2.02] regional accuracy, β= 1.55, SE= 0.20, z= 7.82, p< .001, 95% CI [1.19, 1.97], and an age*displacement error interaction, β= 0.81, SE= 0.16, z= 5.08, p< .001, 95% CI [0.51, 1.15]. There was no statistically significant effect of verbal intelligence, β= 0.21, SE= 0.13, z= 1.65, p= .099, 95% CI [-0.03, 0.48]. The likelihood of correctly generalizing that a baby animal would go the same place as its same-species members depended on children's memory accuracy. Although this contingency existed across the age range, compared to younger children, it strengthened as children's age increased. Again, the contingency of generalization on memory accuracy existed even in younger children, β= 1.14, SE= 0.17, z= 6.70, p< .001, 95% CI [0.41, 1.00].

In the New Species task, we further included category knowledge as another fixed effect given its potential relevance in this particular generalization task. Note that in this model, we calculated the regional accuracy as an average of all same-class species (e.g., mammals: horses, pigs, cows, cats), corresponding to a given species task trial (e.g., generalizing that a new mammal, a deer, would go to Rubyville). Unlike the results in the other generalization

tasks, regional accuracy showed no statistically significant effect on generalization accuracy β= 0.62, SE= 0.13, z= 4.64, p< .001, 95% CI [-0.29, 0.30], was the only significant predictor in the model. Verbal intelligence, β= 0.12, SE= 0.09, z= 1.30, p= .192, 95% CI [-0.06, 0.31], and species class category knowledge, β= -0.12, SE= 0.50, z= -0.24, p= .813, 95% CI [-1.11, 0.87], showed no statistically significant effect on New Species generalization task success.

Supplementary Note 4

Generalization as a Function of Imprecise Regional Accuracy

Next, we tested whether generalization differed as a function of regional accuracy even when children showed unreliable animal-place associative memories, and whether such association depended on age. To this end, we selected generalization trials that were *not* accompanied by perfect regional accuracy for each of the 3 animal exemplars within a given species. For these trials, we calculated whether children had placed 0, 1, or 2 animal exemplars in the correct place, corresponding to 0, 33%, or 66% levels of regional accuracy. We conducted a generalized linear mixed model for the New Animals, New Places, and Baby Animals generalization task separately (see Figure S5), with the following specification:

glmer(generalization accuracy ~ age * regional accuracy count + (1|participant)

For New Animals generalization, we found that generalization accuracy is associated with levels of regional accuracy, β= 0.53, SE= 0.12, z= 4.29, p< .001, 95% CI [0.29, 0.77]. Age showed no statistically significant effect, β= 0.29, SE= 0.17, z= 1.76, p= .079, 95% CI [-0.03, 0.64], and the age * regional accuracy level interaction showed no statistically significant effect, β= 0.12, SE= 0.12, z= 1.03, p= 0.305, 95% CI [-0.11, 0.36] .

For New Places generalization, we found that generalization accuracy is associated with levels of regional accuracy, β= 0.69, SE= 0.12, z= 5.54, p< .001, 95% CI [0.45, 0.93],

which interacted with age, β= 0.32, *SE*= 0.12, *z*= 2.63, *p*= .008, 95% CI [0.08, 0.56]. There was no statistically significant effect of age, β= 0.04, *SE*= 0.16, *z*= 0.28, *p*= .776, 95% CI [-0.27, 0.36].

For Baby Animals generalization, we found that generalization accuracy is associated levels of regional accuracy, β= 0.41, *SE*= 0.12, *z*= 3.39, $p < .001$, 95% CI [0.17, 0.64], which significantly interacted with age, β= 0.33, *SE*= 0.12, *z*= 2.68, *p*= .007, 95% CI [0.09, 0.57]. There was no statistically significant effect of age, β= 0.26, *SE*= 0.15, *z*= 1.79, *p*= .074, 95% CI [-0.02, 0.55].

These analyses showed that for the New Places and Baby Animals tasks, older children are better able to accurately generalize as the level of regional accuracy increases for a given animal species.

Supplementary Tables

Typical	Atypical
**Birds**	
Cardinal	Flamingo
Crow	Pelican
Bluebird	Ostrich
Eagle	Peacock
Parrot	Rooster
Sparrow	Turkey
Hummingbird	Goose
Canary	Quail
**Mammals**	
Cat	Rabbit
Dog	Hippo
Horse	Camel
Lion	Moose
Pig	Sheep
Deer	Squirrel
Cow	Raccoon
Bear	Kangaroo

Table S1. A list of typical and atypical animals within the bird and mammal species classes.

Supplementary Figures

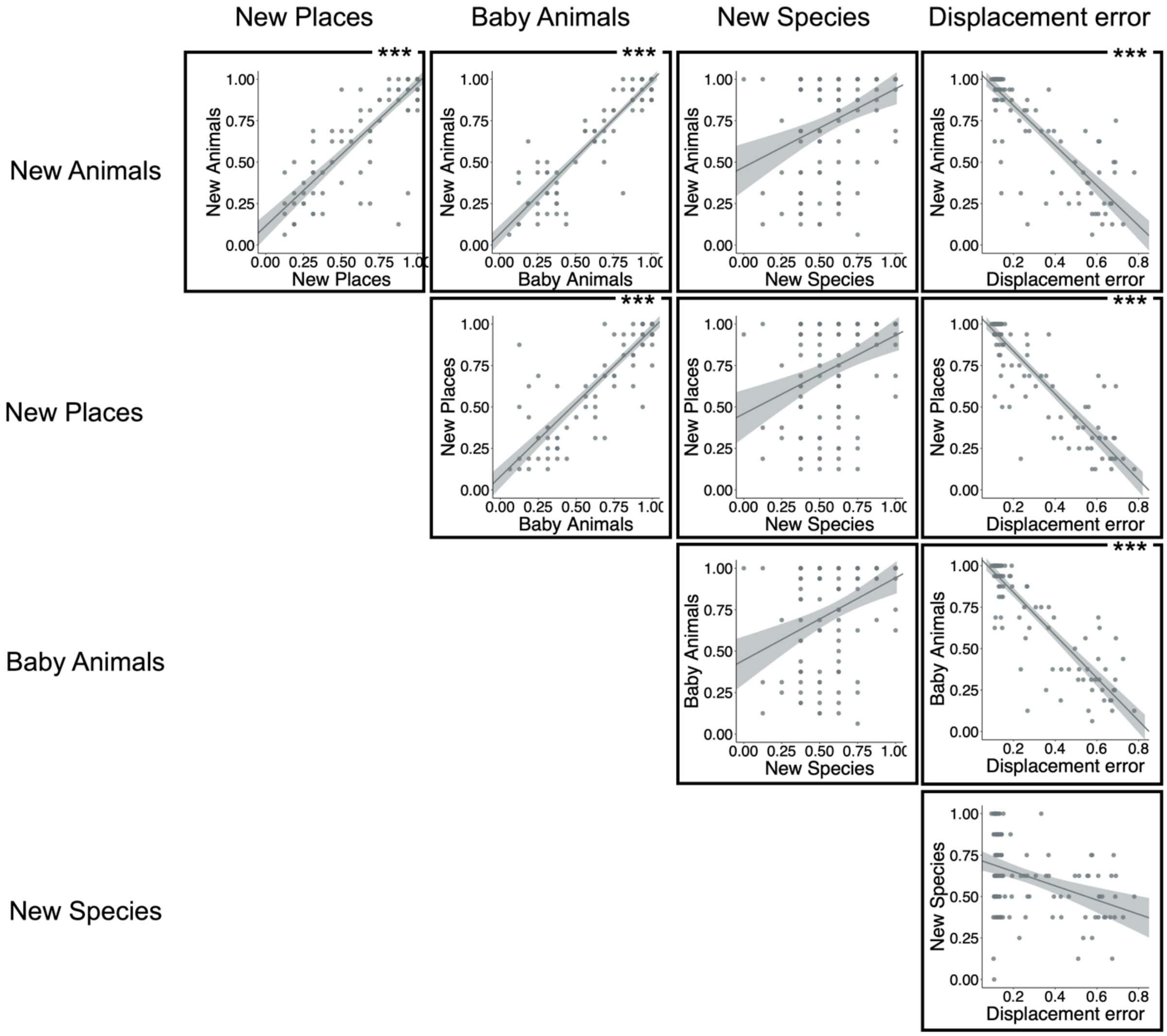

Figure S1. Correlations between Memory Tasks. Scatterplots of the correlations between memory tasks when controlling for age. Significance notation: *p < 0.05; **p < 0.01; ***p < 0.01

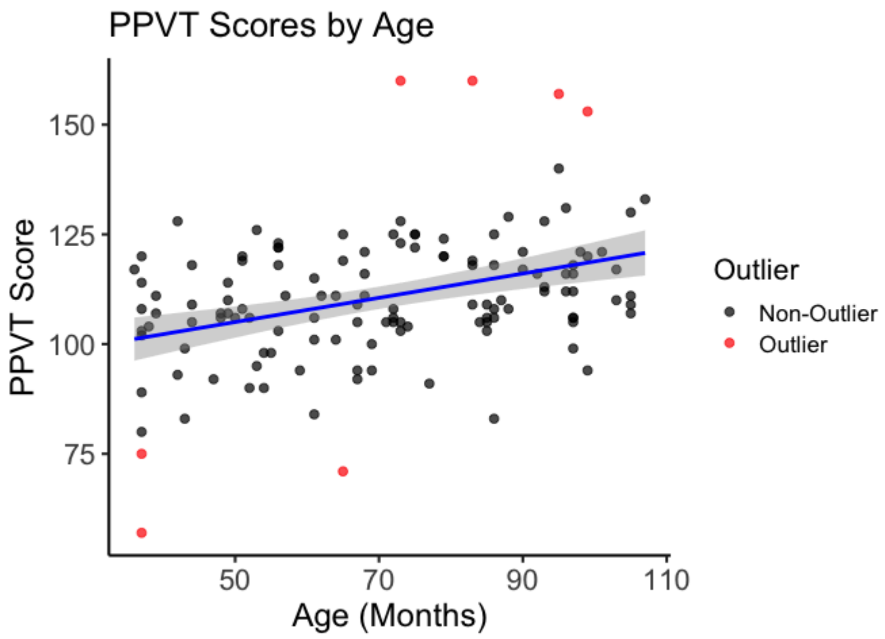

Figure S2. Distribution of Verbal Intelligence Scores. A distribution of PPVT scores (n = 128, M_{PPVT_score} = 110.66, *SD* = 15.57, range = 57-160) (y-axis) against children's age (measured in month; x-axis). Each dot represents a child. Children identified as outliers on PPVT (highlighted in red) were removed in all analyses.

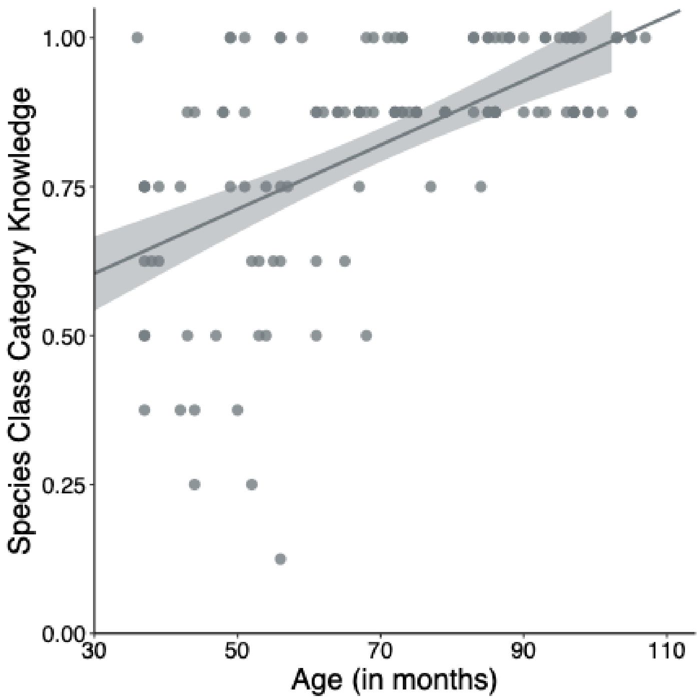

Figure S3. Distribution of Species-Class Category Knowledge by Age. A scatterplot of species-class category knowledge accuracy shown on the *y*-axis, and age (measured in months) on the *x*-axis. Each dot denotes an individual participant. The density of the color indicates the number of participants at a given accuracy level—darker areas reflect more overlapping data points.

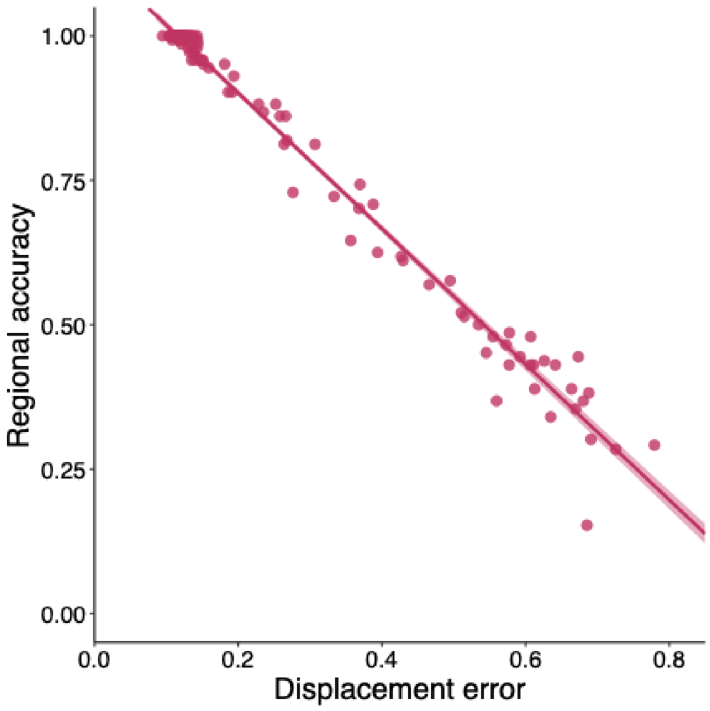

Figure S4. Correlation Between Memory Specificity Indices. A scatterplot of regional accuracy shown on the *y*-axis, and age displacement error on the *x*-axis. Each dot denotes an individual participant. The density of the color indicates the number of participants at a given accuracy level—darker areas reflect more overlapping data points.

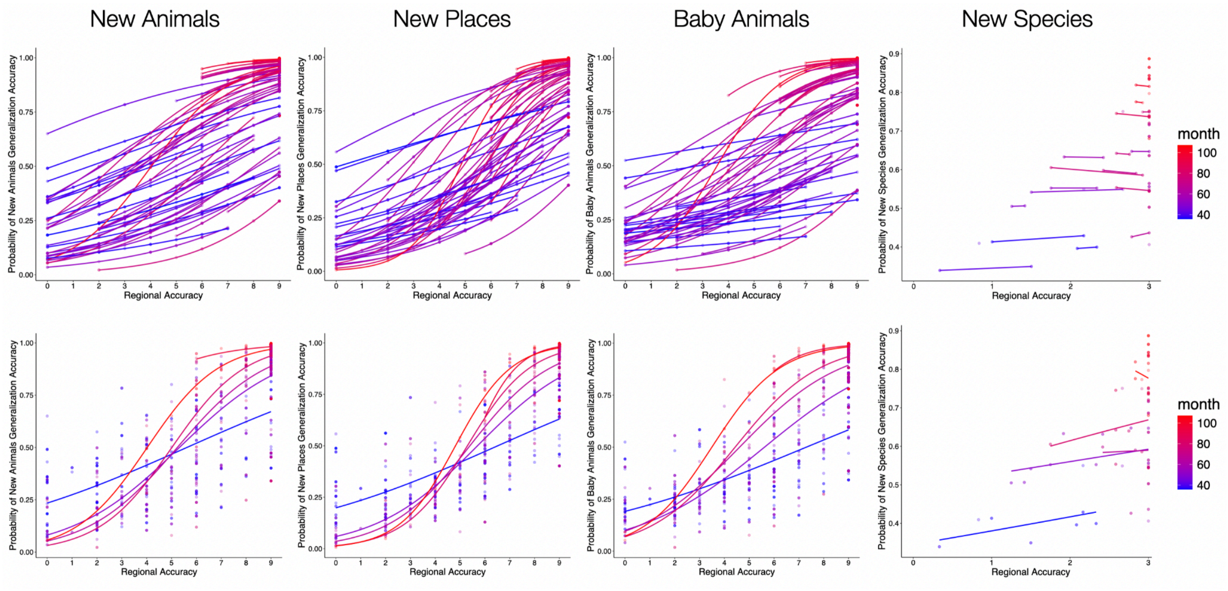

Figure S5. Contingencies of Generalization on Regional Accuracy. Distributions of the estimated probability of generalization accuracy on the trial-by-trial basis (y-axes) by an interaction between regional accuracy (scaled, x-axes) and age in the New Animals (left), New Places (middle), Baby Animals (right) generalization tasks. On the top panel, every line is an individual participant. Color intensity represents age (measured in months). On the bottom panel, participants' ages were grouped only for visualization purposes.

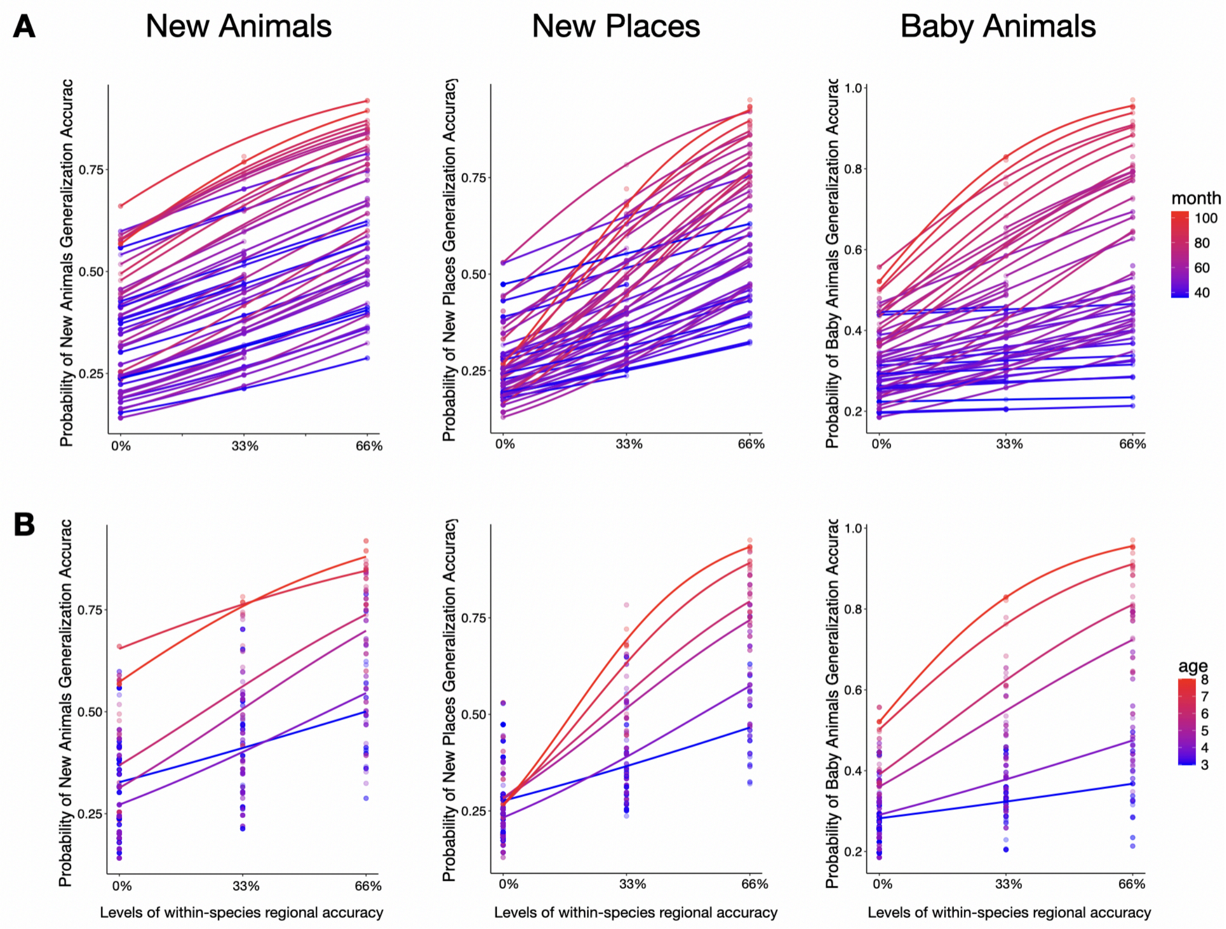

Figure S6. Generalization as a Function of Imprecise Regional Accuracy. Distributions of the estimated probability of generalization accuracy on the trial-by-trial basis (*y*-axes) by an interaction between levels of imprecise memories (scaled, *x*-axes) and age in the New Animals (left), New Places (middle), Baby Animals (right) generalization tasks. Color intensity represents age (measured in months). **(A)** Every line is an individual participant. **(B)** Participants' ages were grouped only for visualization purposes.

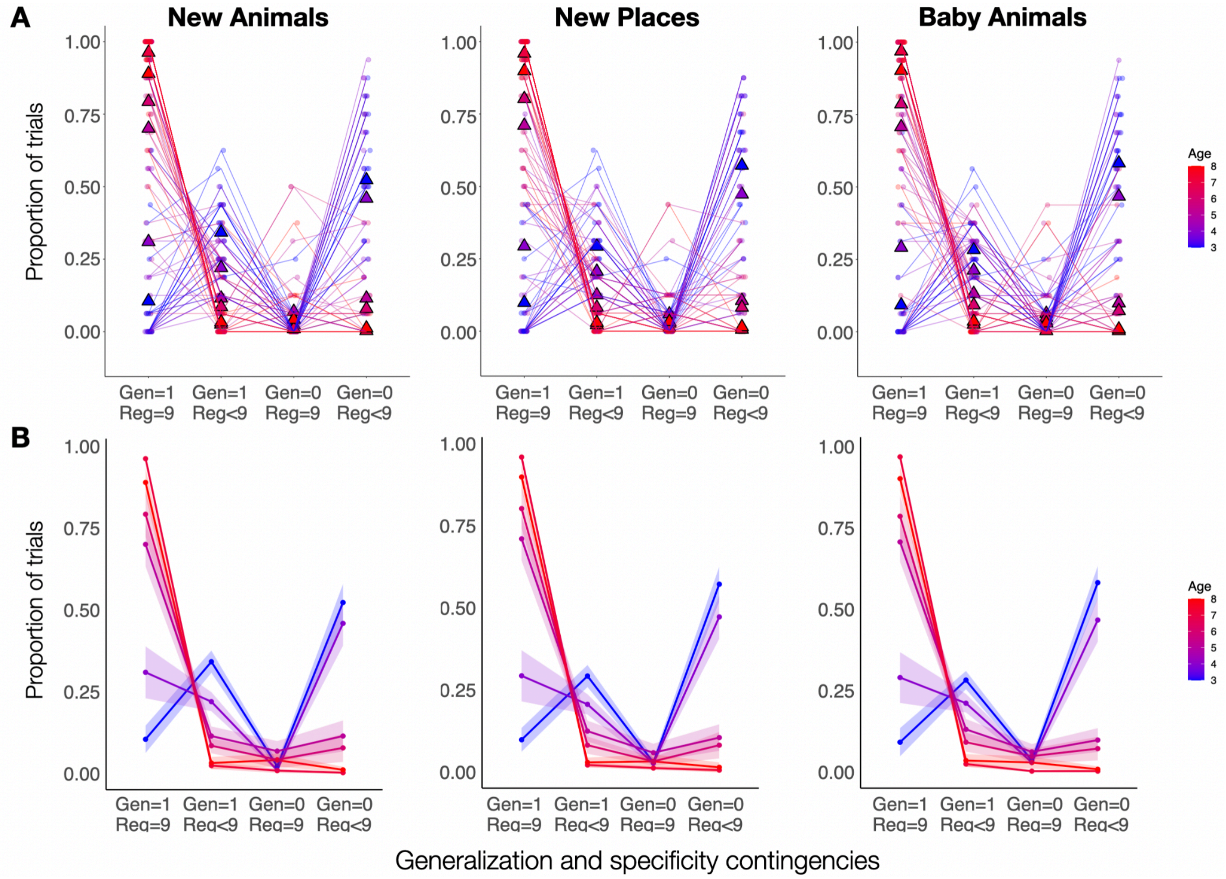

Figure S7. Proportion of trials across the generalization-specificity contingencies. Distributions of the proportion of trials that belong to each of the four generalization – specificity contingency cases: accurate generalization with perfect regional accuracy (denoted as Gen1 & Reg9), imperfect regional accuracy sore (denoted as Gen1 & Reg<9), inaccurate generalization with perfect regional accuracy score (denoted as Gen0 & Reg9), and inaccurate generalization with imperfect regional accuracy score (denoted as Gen0 & Reg<9) in the New Animals, New Places, and Baby Animals generalization tasks. In panel A, each dot represents an individual participant. The triangles represent group-level means separated by age. The group-level means are visualized in panel B for visualization purposes. Shaded ribbons represent standard error per age group. The color spectrum represents children's age.
