# Supplementary material for: The dependence of children’s generalization on episodic memory varies with age and level of abstraction
Source: Nat Commun. 2025 Oct 7;16:8894. doi: 10.1038/s41467-025-63934-w (PMC12504698; doi:10.1038/s41467-025-63934-w)
Supplement: Supplementary file 2 — Reporting Summary [file 41467_2025_63934_MOESM2_ESM.pdf]

nature portfolio

Corresponding author(s): Sabrina Karjack

Last updated by author(s): Jul 18, 2025

Reporting Summary

Nature Portfolio wishes to improve the reproducibility of the work that we publish. This form provides structure for consistency and transparency in reporting. For further information on Nature Portfolio policies, see our Editorial Policies and the Editorial Policy Checklist.

Statistics

For all statistical analyses, confirm that the following items are present in the figure legend, table legend, main text, or Methods section.

n/a	Confirmed	
☐	☒	The exact sample size (*n*) for each experimental group/condition, given as a discrete number and unit of measurement
☐	☒	A statement on whether measurements were taken from distinct samples or whether the same sample was measured repeatedly
☐	☒	The statistical test(s) used AND whether they are one- or two-sided<br>*Only common tests should be described solely by name; describe more complex techniques in the Methods section.*
☐	☒	A description of all covariates tested
☐	☒	A description of any assumptions or corrections, such as tests of normality and adjustment for multiple comparisons
☐	☒	A full description of the statistical parameters including central tendency (e.g. means) or other basic estimates (e.g. regression coefficient) AND variation (e.g. standard deviation) or associated estimates of uncertainty (e.g. confidence intervals)
☐	☒	For null hypothesis testing, the test statistic (e.g. *F*, *t*, *r*) with confidence intervals, effect sizes, degrees of freedom and *P* value noted<br>*Give P values as exact values whenever suitable.*
☐	☒	For Bayesian analysis, information on the choice of priors and Markov chain Monte Carlo settings
☐	☒	For hierarchical and complex designs, identification of the appropriate level for tests and full reporting of outcomes
☐	☒	Estimates of effect sizes (e.g. Cohen's *d*, Pearson's *r*), indicating how they were calculated

Our web collection on statistics for biologists contains articles on many of the points above.

Software and code

Policy information about availability of computer code

Data collection	Data was collected using both Qualtrics (2023) and PsychoPy 3.0.0. (2019). The code for PsychoPy task is publicly available on OSF [https://doi.org/10.17605/OSF.IO/2GAHZ]
Data analysis	All statistical analyses were conducted in R 4.1.2 (R Core Team, 2021) using RStudio 2022.07.2 Build 576 (RStudio Team, 2022). The code used to analyze the data is publicly available on OSF [https://doi.org/10.17605/OSF.IO/2GAHZ]

For manuscripts utilizing custom algorithms or software that are central to the research but not yet described in published literature, software must be made available to editors and reviewers. We strongly encourage code deposition in a community repository (e.g. GitHub). See the Nature Portfolio guidelines for submitting code & software for further information.

Data

Policy information about availability of data

All manuscripts must include a data availability statement. This statement should provide the following information, where applicable:

- Accession codes, unique identifiers, or web links for publicly available datasets
- A description of any restrictions on data availability
- For clinical datasets or third party data, please ensure that the statement adheres to our policy

Data are publicly available through the Open Science Framework at [https://doi.org/10.17605/OSF.IO/2GAHZ].

Research involving human participants, their data, or biological material

Policy information about studies with human participants or human data. See also policy information about sex, gender (identity/presentation), and sexual orientation and race, ethnicity and racism.

Reporting on sex and gender	Participants' sex and gender were reported by a parent or guardian. As reported in the manuscript, the sample included 131 children aged 3-8 years (70 female; 59 male; 1 non-binary/prefer not to say; 1 transgender female; Mmonth = 70.07, SD = 21.21, range = 36-107). There were no statistically significant sex differences on any of the generalization tasks, as reported in the results section of the manuscript.
Reporting on race, ethnicity, or other socially relevant groupings	Participants' parents/guardians provided general demographic information including information on race, and socioeconomic status, including parental education, household income, and occupation. We did not have any predictions with respect to the effect of race and other socially-relevant groupings on memory performances.
Population characteristics	As stated above, our sample included 131 children aged 3-8 years (70 female; 59 male; 1 non-binary/prefer not to say; 1 transgender female; Mmonth = 70.07, SD = 21.21, range = 36-107).
Recruitment	Participants were recruited from Philadelphia and the surrounding suburbs through a Temple University database of families who had expressed interest in participating in research, online advertisements, and community recruitment at events such as farmers markets. There are no potential self-selection or other biases that may have impacted recruitment or results. This was a convenience sampling approach. The sample was representative of the area and selected population.
Ethics oversight	The Temple University Institutional Review Board approved the study protocol.

Note that full information on the approval of the study protocol must also be provided in the manuscript.

Field-specific reporting

Please select the one below that is the best fit for your research. If you are not sure, read the appropriate sections before making your selection.

☐ Life sciences ☒ Behavioural & social sciences ☐ Ecological, evolutionary & environmental sciences

For a reference copy of the document with all sections, see nature.com/documents/nr-reporting-summary-flat.pdf

Behavioural & social sciences study design

All studies must disclose on these points even when the disclosure is negative.

Study description	This is a quantitative experimental design.
Research sample	A total sample of 131 children aged 3-8 years (70 female; 59 male; 1 non-binary/prefer not to say; 1 transgender female; Mmonth = 70.07, SD = 21.21, range = 36-107) recruited from Philadelphia and the surrounding suburbs participated in the study. The sample is a convenience sample representative of the Philadelphia metropolitan area and surround suburbs.
Sampling strategy	The sample is a convenience sample. We did not conduct any power analyses prior to data collection. Our sample size was determined based on previous studies with similar age ranges (ages 4 to 8) that detected robust age-related differences in memory specificity (Ngo, Newcombe, & Olson, 2019, Child Development) and memory generalization (Ngo, Benear, Popal, Olson, & Newcombe, 2021, Current Biology). In these studies, there were aprroximately 20-25 children per age group. In this study, we aimed for approximately 22 children per age group.
Data collection	Data was collected using a laptop computer and pen and paper. Only the researcher and participant were in the experimental room. The researcher was not blind to the condition or study hypotheses.
Timing	Data collection ran from 03/20/2022 until 10/16/2022. There was no gap in data collection.
Data exclusions	Seven participants were identified as outliers on the Peabody Picture Vocabulary Test 5th Edition and therefore were excluded from all analyses.
Non-participation	One child did not complete the experiment and two children did not complete the Peabody Picture Vocabulary Test (PPVT), resulting in a sample size of 128 children who completed the full procedure.
Randomization	Participants were randomly assigned to the different counterbalancing task versions.

Reporting for specific materials, systems and methods

We require information from authors about some types of materials, experimental systems and methods used in many studies. Here, indicate whether each material, system or method listed is relevant to your study. If you are not sure if a list item applies to your research, read the appropriate section before selecting a response.

Materials & experimental systems

n/a	Involved in the study
☒	☐ Antibodies
☒	☐ Eukaryotic cell lines
☒	☐ Palaeontology and archaeology
☒	☐ Animals and other organisms
☒	☐ Clinical data
☒	☐ Dual use research of concern
☒	☐ Plants

Methods

n/a	Involved in the study
☒	☐ ChIP-seq
☒	☐ Flow cytometry
☒	☐ MRI-based neuroimaging

Plants

Seed stocks

Report on the source of all seed stocks or other plant material used. If applicable, state the seed stock centre and catalogue number. If plant specimens were collected from the field, describe the collection location, date and sampling procedures.

Novel plant genotypes

Describe the methods by which all novel plant genotypes were produced. This includes those generated by transgenic approaches, gene editing, chemical/radiation-based mutagenesis and hybridization. For transgenic lines, describe the transformation method, the number of independent lines analyzed and the generation upon which experiments were performed. For gene-edited lines, describe the editor used, the endogenous sequence targeted for editing, the targeting guide RNA sequence (if applicable) and how the editor was applied.

Authentication

Describe any authentication procedures for each seed stock used or novel genotype generated. Describe any experiments used to assess the effect of a mutation and, where applicable, how potential secondary effects (e.g. second site T-DNA insertions, mosiacism, off-target gene editing) were examined.
